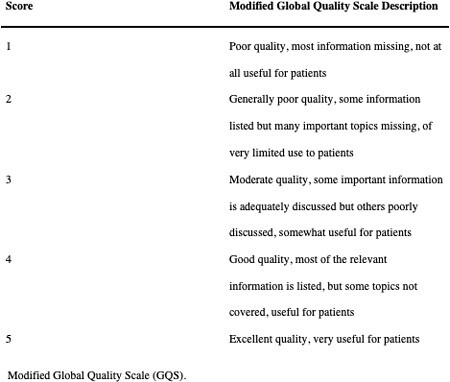# 573 Comparing ChatGPT and Google in Burn Injury Education: Answering Common Burn Injury and Management Questions

**DOI:** 10.1093/jbcr/irae036.207

**Published:** 2024-04-17

**Authors:** Jose Antonio Arellano, Sumaarg Pandya, Mario Alessandri-Bonetti, Hilary Liu, Tiffany Jeong, Jenny A Ziembicki, Francesco Egro

**Affiliations:** University of Pittsburgh Medical Center, Pittsburgh, PA; University of Pittsburgh Medical Center, Pittsburgh, PA; University of Pittsburgh Medical Center, Pittsburgh, PA; University of Pittsburgh Medical Center, Pittsburgh, PA; University of Pittsburgh Medical Center, Pittsburgh, PA; University of Pittsburgh Medical Center, Pittsburgh, PA; University of Pittsburgh Medical Center, Pittsburgh, PA

## Abstract

**Introduction:**

In the era of digital information, patients often use Google to find answers to their questions on medical procedures. With the emergence and increasing popularity of artificial intelligence (AI) chatbots, patients may turn to such technologies, such as ChatGPT, as an alternative source of medical information. This study aims to determine whether ChatGPT provides safe, accurate, and comprehensive medical answers to the most commonly asked questions regarding burn injury and their management by comparing the quality of answers provided by ChatGPT and Google search.

**Methods:**

A Google search was conducted using the search term “burn.” The ten most searched questions and the answers provided by Google were recorded. The same questions were asked to ChatGPT. The chat history was cleared after each query to minimize the impact of previous responses. Three surgeons with over 10 years of experience in burn trauma and treatment were then asked to grade the quality of the answers provided by Google and ChatGPT from 1 (poor quality) to 5 (excellent quality) according to the Global Quality Score (GQS). Respondents were also asked for each question which source they would prefer their patients get information from. Respondents were blinded to the information sources. A Wilcoxon paired t-test was performed to evaluate the difference in GQS ratings for Google and ChatGPT answers.

**Results:**

The average score for answers provided by Google was 2.80 ± 1.03, indicating that some information was present but important topics were missing. Conversely, the average score for ChatGPT-generated answers was 4.57 ± 0.73, indicating that it provided excellent quality responses that were highly useful to patients. For half of the questions, respondents unanimously preferred for their patients to receive their information from ChatGPT. For the remaining five questions, two surgeons chose ChatGPT while one preferred Google.

**Conclusions:**

This study presents an initial comparison of Google and ChatGPT responses for commonly asked burn injury questions, highlighting their respective strengths and limitations. Based on the feedback of three experienced surgeons, ChatGPT outperforms Google in providing high-quality patient education to commonly asked questions on burn injury and management. These results highlight the potential of AI as a superior source of patient education.

**Applicability of Research to Practice:**

Further research is warranted to validate these findings on a larger and more diverse dataset and to assess the potential of combining ChatGPT with traditional web search mediums. The integration of AI technology with accessible and familiar search engines has the potential to optimize patient queries on burn questions, leading to improved patient education and outcomes. This study provides validation that ChatGPT is a useful tool for patient education and commonly asked medical questions. However, its utility has yet to be determined in a clinical setting for medical decision-making.